# Occlusal splint effects on visual capacities in patients with temporomandibular disorders (TMD): a prospective interventional cohort study

**DOI:** 10.1038/s41405-025-00337-5

**Published:** 2025-06-09

**Authors:** Shahnawaz Khijmatgar, Gianluca Martino Tartaglia, Andrea Sardella, Alessandro Marchesi, Roberto Marchesi, Clarita Pellegrini

**Affiliations:** 1 Department of Biomedical, Surgical and Dental Sciences, University of Milan, Milan, Italy; 2 Ospedale Maggiore Policlinico, UOC Maxillo-Facial Surgery and Dentistry, IRCCS Cà Granda, Milan, Italy; 3 Department of Gnathology, ASST Santi Paolo e Carlo, University of Milan, Milan, Italy; 4 Department of Ophthalmology, Vizzolo Predabissi Hospital, Milan, Italy

**Keywords:** Malocclusion, Oral pathology

## Abstract

**Objectives:**

The temporomandibular joint system and visual apparatus seem to be correlated. Our study aimed to examine the potential effects of occlusal splints on visual capacities (accommodation and ocular convergence) in individuals with temporomandibular disorders, followed for a period of 6 months and 1 year, assessing changes over this timeframe.

**Materials and methods:**

Forty-two subjects were enrolled in a year-long study conducted at the Operative Unit of Odontostomatology of ASST Santi Paolo e Carlo, in collaboration with the University of Milan, Italy. A gnathological examination was followed by an orthoptic assessment using the stick of Duane and measuring convergence and accommodation at three jaw positions at different time points (T0, T1, T2, T3).

**Results:**

After 6 months of occlusal splint therapy, an improvement in visual abilities at maximum intercuspation and resting positions was observed. In contrast, the open-mouth position did not yield statistically significant results. Further assessments at 1 year did not show significant changes. Occlusal splint therapy appears to positively influence visual capacities (in maximum intercuspation and resting positions). While the open-mouth position did not exhibit significant improvements.

**Conclusion:**

Our study results highlight the importance of considering jaw positions in evaluating visual function, suggesting the possible integration of occlusal splints with an orthoptic assessment in comprehensive TMD management.

## Introduction

Temporomandibular disorders (TMDs), orthodontic, and ophthalmic problems appear to be closely connected. The connection between these systems involves three types: 1. anatomical, through craniofacial sutures; 2. neurological, through links between brain nuclei like vestibular, trigeminal, oculomotor, and accessory nuclei; and 3. functional, through muscle chains [1, 2].

Valesan et al. [3] conducted a systematic review and meta-analysis, finding that the prevalence of temporomandibular joint (TMJ) disorders (TMD) ranges from 4.6% to 37.1% across different studies, with a higher prevalence in females and individuals aged 20–40 years. This aligns with the general consensus that TMD affects 5–12% of the general population, with a female predominance.

Trigeminal nerve (TN) is the fifth and largest cranial nerve, and it is considered as both sensory and motor nerve. It supplies the head and neck region that includes ophthalmic, maxilla, and mandibular region,s and these areas are supplied through the ophthalmic, maxilla, and mandibular divisions of TN. The existence of a possible relationship between the stomatognathic and visual apparatus is principally based on a neuroanatomical connection between the two systems: the idea is that some afferents from periodontal receptors, through the sensory and midbrain nuclei of the TN and the medial longitudinal fasciculus, arrives to the motor nuclei of some cranial nerves (in particular oculomotor, trochlear, abducens and accessory nerves) In addition, from these nucleus some fibers go to mechanoreceptors for regulation of bite force and to oculomotor nerves for ocular muscle spindles.

Also, the trigeminal midbrain nucleus appears to be connected with another anatomical structure called the superior colliculi, which have a sensorimotor integration function. They are also involved in processing optical stimuli, orienting attention, and coordinating eye and head movements [4, 5].

Also, the main sensory receptors in the head-neck region are the stomatognathic/TMJ and oculomotor systems [6], which can impact patients’ balance and behavior [7].

However, these neuroanatomical explanations seem to be a still limited exploration of the relationship between TMDs and vision issues. Some studies by Monaco and colleagues in 2003 and 2006 revealed that jaw disfigurement and myofascial pain can reduce eye convergence [8–11]; more precisely, Monaco [8] studied the association between ocular convergence defects (OCD) and TMDs in 48 adult subjects with TMD-related symptoms and 48 control subjects. Ocular convergence was assessed using two tests. The first test revealed that 75% of the TMD group exhibited compromised convergence, with 36% falling in the 5–7 cm range and 48% in the >7 cm range. The second test, i.e., Berens prism test, showed 28% with <18 diopters and 72% with 18–25 diopters in the TMD group, significantly higher than the 21% in the control group. TMD patients reported specific symptoms such as limited maximal opening, myofascial pain, headaches, and neck stiffness, which were more prevalent in individuals with compromised convergence. The study concluded a higher prevalence of OCD in TMD patients experiencing head, neck, and shoulder pain [8–12].

Cuccia and Caradonna found reduced binocular vision in patients with TMJ disc disorders [13]. Monaco et al. [8], Caruso [14], and Vompi [8–14, 15, 16] investigated associations between OCD, TMDs, and dental occlusion dysfunctions. Caruso [14] cross-sectional study aimed to investigate correlations between visual impairments and dental occlusion dysfunctions. The test group comprised 34 subjects (21 males, 13 females, mean age 11 ± 2 years) meeting specific inclusion criteria related to visual and craniofacial health.

Visual clinical tests were used to identify problems on how the eye vision work together and their ability to focus. Orthodontic examinations included occlusal molar relationships as one of the key indicators and primary endpoints. The statistical association demonstrated that there was a significant association between occlusal molar relationships and specific eye movement, i.e., exodeviations. Additionally, people with a certain type of bite (class-II molar relationship) were more likely to have weaker eye coordination than expected [14, 15, 17].

Vompi C. 2020 [15] study aimed to explore the relationship between TMDs, orthodontic diseases, and vision dysfunctions by evaluating the prevalence and distribution of vision defects in patients with TMD. The study included *n* = 100 TMD patients, and various outcomes, including gnathological parameters, occlusal and skeletal parameters, and orthoptic parameters, were considered. The study compared the frequency of vision defects in the sample with the average frequency in the Italian population. The study results found a higher frequency of refractive defects in the sample included, with phorias being the most common (92%). Significant associations were observed between ocular convergence reduction and disc displacement with reduction, as well as between asymmetry and motor ocular deviations [15, 17–19].

Baldini [20] review focuses on the relationship between visual functions, specifically ocular disorders, and the stomatognathic apparatus (related to the mouth and jaws). The developmental plasticity of visual functions in early postnatal life, which involves the oculomotor system, suggests potential correlations between vision and dental occlusion. However, the scientific evidence supporting this connection is not well-established. The review analyzes clinical data collected from major databases, revealing a middle level of evidence primarily derived from case-control and cross-sectional studies. While there is evidence of a correlation between ocular disorders and dental occlusion, such as myopia, hyperopia, astigmatism, exophoria, and gait issues due to convergence defects, establishing a cause-effect relationship remains uncertain. The conclusion suggests the need for future studies with higher levels of evidence, including prospective, controlled, and randomized studies, to better understand this correlation [20].

Milani et al. tried to evaluate the accommodation ability in a group of athletes after the use of an occlusal splint called Mandibular Orthopedic Repositioning Appliances [21].

Finally, another study tried to investigate possible changes on visual abilities after a treatment with a tongue elevator called ELIBa; it’s used, for example, in subjects suffering from atypical swallowing to modify the swallowing pattern during sleep and consolidate the effects obtained with myofunctional and speech therapy [22, 23]. These studies hint at an association between ocular issues, dental occlusion, and TMD, but the evidence is limited, and further robust research is required to establish causation and the mechanisms involved.

So, since the existing literature suggests the potential impact of TMDs on vision [24], we aim to identify if an occlusal splint treatment (the most common method to manage TMD disorders) in individuals with TMDs could influence visual function, specifically the accommodative capacity and ocular convergence. The rationale for this hypothesis is from the complex interdependence between the TMJ and the visual apparatus, as evidenced by neural connections between extraocular muscles, periodontal receptors, dental pulp, masticatory muscles, and TMJ receptors. More precisely, our research question was: “Does occlusal splint treatment, exert measurable effects on the accommodative capacity and ocular convergence in individuals with TMDs, thereby providing insights into the dynamic interplay between the TMJ and the visual apparatus?”

The temporal trajectory of our investigation spans from T0, representing the commencement of occlusal splint treatment, to T2, a pivotal time point 6 months post-initiation, and T3 (after 1 year from T0 but only for nine patients). Through meticulous assessments at these endpoints, we seek to delineate dynamic alterations in visual parameters, contributing to a nuanced understanding of interconnected dynamics between the TMJ and the visual apparatus in TMD patients.

## Materials and methods

### Study design

A prospective interventional cohort study was designed. The duration of follow-up was over 6 months who were suffering from TMJ disorders (TMDs). The study was conducted as per the Helsinki Declaration, and ethical approval was sought from the institutional review board of the University of Milan, Italy (SCOTMJI/2022). Informed consent was obtained from all the participants for participation in the study and dissemination of anonymous data for scientific publication.

### Study settings

The study was performed at the Operative Unit of Odontostomatology of ASST Santi Paolo e Carlo, associates of the University of Milan, during a 1-year period on a sample of 42 subjects (30 females and 12 males).

### Inclusion criteria

Both female and male patients under 55 years old with TMD, in particular bruxism (clenching or grinding), muscle contracture, reducible and non-reducible disc dislocation, and that who needed treatment with an occlusal splint, with or without refractive defects (in particular myopia and astigmatism) were included.

### Exclusion criteria

Subjects over 55 years old were excluded from the study cause the physiological decline in visual capacity were excluded. Then we excluded patients with arthrosis or who needed a treatment with infiltration of hyaluronic acid, but also subjects with ocular diseases, congenital ophthalmopathies, or facial trauma.

The subjects underwent first a gnathological examination based on anamnesis, physical examination, and, where necessary, some imaging techniques such as orthopantomography, TMJ stratigraphy, and nuclear magnetic resonance imaging of the TMJ were used in order to understand the type of TMD. Subsequently, the subjects underwent the visual tests: the “convergence test” and the “accommodation test,” that were carried out allowing subjects with refractive defects to wear glasses or contact lenses. This made it possible to consider all patients as emmetropic. The orthoptic examinations were performed in three different jaw positions: maximum intercuspation (A1 and C1), resting position (A2 and C2) and open mouth (A3 and C3), in different times: T0(at the beginning of the occlusal splint treatment), T1 (after 3 months from T0), T2 (after 6 months from T0) and T3 after 1 year from T0 (but only for nine patients) to understand whether the treatment could have any influence on visual ability in terms of improvement or deterioration. We have to specify that the maximum intercuspation refers to the position of the mandible when teeth are brought into full interdigitation with the maximal number of teeth contacting. While in the resting position, the teeth are not in contact and the lips rest gently upon each other.

We decided to reevaluate patients at three and 6 months because occlusal splints therapy normally requires a period from 3 to 6 months for the active phase, and then for another 6 months for maintenance [12–27].

**Fig. 1:**
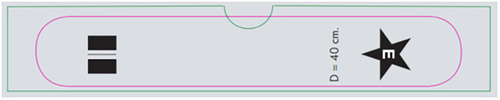
“Stick” of Duane’s test.

For the orthoptic examination for both convergence and accommodation tests, we used a fixation target called the stick of Duane’s test—Fig. 1. With the convergence test, we tried to understand the possible neuromuscular disorganization of the extraocular muscle tone. So we started with the operator sat down in front of the tested subject who focused on the star with the central E positioned at the midline level of the face and about 40 cm from the root of the nose; then the fixation target is brought towards the tip of the nose and after using, as a reference point, the outer canthus of the eye of the patient tested, we measured with a ruler the distance at which one eye deviates laterally since it can no longer maintain binocular vision and consequent convergence. This distance represents the near point of convergence, which is the point where the eye deviates laterally, and it’s called the “breakpoint.” We considered the normal values of convergence described in Cooper and Jamal’s Major Review, which classifies as hypo-convergent all the subjects who presented a “breakpoint” greater than 7.5 cm [28]. For simplicity in our study, we only considered the breaking point, but a complete examination of the oculomotor function should also evaluate other parameters.

For the accommodation test, we used the opposite pole of the “stick” of Duane’s test, represented by the thin vertical line placed in the middle of the two black rectangles. The tested subject focused on the line positioned about 40 cm from the root of the nose and brought close to the tip of the nose; then we asked the subject to report the first signs of “blurring” of the central vertical line or its disappearance. With a ruler, we measured the distance at which the “blurring” occurs. It’s called the near point of accommodation (NPA) which is the closest point that can be seen clearly with accommodation at its maximum. The farthest point at which an object can be seen clearly with accommodation at its lowest value is called the far point of accommodation (FPA). This value varies according to refractive status (ametropia).

Using the Donders formula: AA = (NPA)−(FPA), we could calculate the amplitude of accommodation (AA), which represents the variation of accommodative power that the crystalline lens is capable of performing. In our study, we treated all subjects as emmetropes, so we could set the FPA point at infinity. In this way, the value of the NPA corresponds to the value of the AA. The results measured in centimeters were firstly converteted in meters and then transformed into diopters by calculating the inverse of the distance; this allowed the comparison between the AA found and the average amplitude of accommodation derived empirically on the basis of the age variable only using the Hofstetter formula in which the AA is (AA = 15−0.25 × age). In this way, we found the value of AA that a certain individual should have at a specific time of life: if the AA defined by the Donders formula was lower than the average amplitude of accommodation, the subject was to be categorized as “pathological” for the accommodative variable [4].

With regard to accommodation, a higher value measured indicates a lower accommodative capacity, and a higher convergence value indicates a lower neuromuscular capacity. To make the difference between blurring and double vision of the fixation target more apparent to the test subjects, the accommodation test was carried out in monocular vision (conventionally, we chose the right eye for all patients), while the convergence test was performed in binocular vision (a necessary condition).

### Statistical analysis

A descriptive statistics summarizing baseline characteristics at T0. Means, standard deviations, 95% CI, and ranges were calculated for continuous variables, while frequencies and percentages were provided for categorical variables, such as gender and severity of TMD symptoms. Subsequent assessments included tests for significant differences or imbalances between groups. Normal distribution of the data was analyzed. Paired sample t-tests were employed to assess changes in accommodative capacity and ocular convergence within each group from T0 to T2, and Wilcoxon Signed-Rank tests were utilized for non-normally distributed data. Independent sample *t*-tests or Mann–Whitney *U* tests were conducted to compare changes between groups, specifically the occlusal splint treatment group and any control or comparison group.

The Random Forest model was used to analyze the data, and its performance was measured using two key values, i.e., mean squared error (MSE) and *R*-squared (*R*²) value. The purpose of the analysis was to evaluate whether occlusal splint therapy (OST) affects accommodation and ocular convergence in individuals with TMDs. By applying machine learning models (Random Forest), the goal was to determine if changes in jaw position and occlusal treatment have a measurable impact on eye function over time. If the results are found to be valid, this could lead to new diagnostic and treatment strategies that integrate dental, visual, and neurological health for better patient outcomes.

## Results

First, we calculated the average of the measurements and we observed that the values of accommodation and oculomotor function showed a worsening tendency when the test was carried out in the open mouth position (Table 1).

**Table 1:** Descriptive statistics.

Variable	Observation	Mean	Std. dev.	SE	Min	Max	95% CI
C1	126	8.972222	2.156206	0.1920901	5	15	8.59, 9.35
C2	126	9.063492	2.115641	0.1884763	5	14.5	8.69, 9.43
C3	126	10.10317	2.094104	0.1865576	6	16.5	9.73, 10.47
A1	126	12.03968	2.649229	0.2360121	6.5	22.5	11.57, 12.50
A2	126	11.84127	2.700112	0.2405451	6	19.5	11.36, 12.31
A3	126	12.99603	2.782442	0.2478796	7	21	12.50, 13.48
Age	126	30.52381	10.86478	0.9679115	15	55	28.60, 32.43

Later, we compared the values from T0 to T2 in the three different mandibular positions and obtained a better ocular convergence after 6 months of treatment with an occlusal splint in the maximum intercuspation and rest position. While in the open mouth position, there were no didn’t have statistical significance results.

Lastly, only 9 subjects of the 42 included in our study performed the vision tests also after 1 year from T0, but didn’t find any statistically significant results, probably due to the small sample size.

- Time Points (*t*): The dataset contains equal numbers of records for each time point (T0, T1, T2).
- Convergence Test (C1, C2, C3) and Accommodation Test (A1, A2, A3): These columns show the measurements for each test in different jaw positions. The means, standard deviations, and range (min to max) give an idea of the spread and central tendency of these measurements.
- Gender: There are 90 females and 36 males in the study.
- Age: The age of participants ranges from 15 to 55 years, with a mean age of ~30.5 years.

### Convergence test (C)

Jaw Position 1: *F* value = 3.47, *P* value = 0.034

Jaw Position 2: *F* value = 1.01, *P* value = 0.367

Jaw Position 3: *F* value = 1.50, *P* value = 0.228

### Accommodation test (A)

Jaw Position 1: *F* value = 0.98, *P* value = 0.376

Jaw Position 2: *F* value = 1.73, *P* value = 0.181

Jaw Position 3: *F* value = 0.36, *P* value = 0.699

The only statistically significant result (*P* value < 0.05) is observed in the Convergence Test at Jaw Position 1, indicating that there are significant differences in measurements across time points (T0, T1, T2) for this specific test and position.

For all other test types and jaw positions, the *P* values are above 0.05, suggesting that the differences in measurements across time points are not statistically significant for those categories.

The Two-sample Wilcoxon rank-sum (Mann–Whitney) tests were conducted to compare the outcomes between male and female groups. The results indicated that there were no significant differences between the two groups for any of the variables tested. Specifically, the *p* values for all outcomes were greater than 0.05, indicating that the differences observed were not statistically significant. This suggests that the distributions of the variables C1, C2, C3, A1, A2, and A3 were similar between males and females in the study sample (Tables 2 and 3).

**Table 2:** Gender distribution.

Gender	Freq.	Percent	Cum.
Male	36	28.57	28.57
Female	90	71.43	100
Total	126	100

**Table 3:** Test for different outcomes based on gender.

Outcomes	Observations	Mean	SE	SD	95% CI	*p* value
Male						
C1	36	9.541667	0.355442	2.132654	8.82008–10.26325	0.01
C2	36	9.597222	0.352328	2.113965	8.881959–10.31249	0.01
C3	36	10.43056	0.386864	2.321184	9.64518–11.21593	not significant
A1	36	12.625	0.521892	3.131351	11.5655–13.6845	not significant
A2	36	12.20833	0.515388	3.092329	11.16204–13.25463	not significant
A3	36	13.65278	0.549827	3.29896	12.53657–14.76899	not significant
Female						
C1	90	8.744444	0.225032	2.134843	8.29731–9.191579	0.01
C2	90	8.85	0.220295	2.089903	8.412278–9.287722	0.01
C3	90	9.972222	0.210279	1.994883	9.554402–10.39004	not significant
A1	90	11.80556	0.254018	2.409828	11.30083–12.31028	not significant
A2	90	11.69444	0.266725	2.530371	11.16447–12.22442	not significant
A3	90	12.73333	0.265611	2.519809	12.20557–13.2611	not significant

### Correlation analysis

#### Age

There seems to be a very low correlation between age and the measurements. This suggests that the age of the subjects doesn’t have a strong linear relationship with the outcomes of the convergence and accommodation tests (Table 2).

#### Gender

The correlation between gender and measurements is also quite low, indicating that gender does not have a significant linear impact on the test results (Table 2).

Measurement values: as expected, there’s no correlation between the measurements and the categorical variables like test type and jaw position, as these are distinct test categories and positions.

Test type and jaw position: there’s no significant correlation between these variables and the measurements, which aligns with the distinct nature of these test categories.

Considering the results from both the ANOVA and correlation analysis, we can conclude that while there are some significant differences in convergence measurements at Jaw Position 1 across different time points, overall, the influence of age, gender, and jaw position on the test outcomes is not strongly linear. Further analysis, potentially involving more complex statistical models or considering non-linear relationships, could provide additional insights.

#### Correlation matrix of variables related to occlusal splint treatment outcomes

**Fig. 2:**
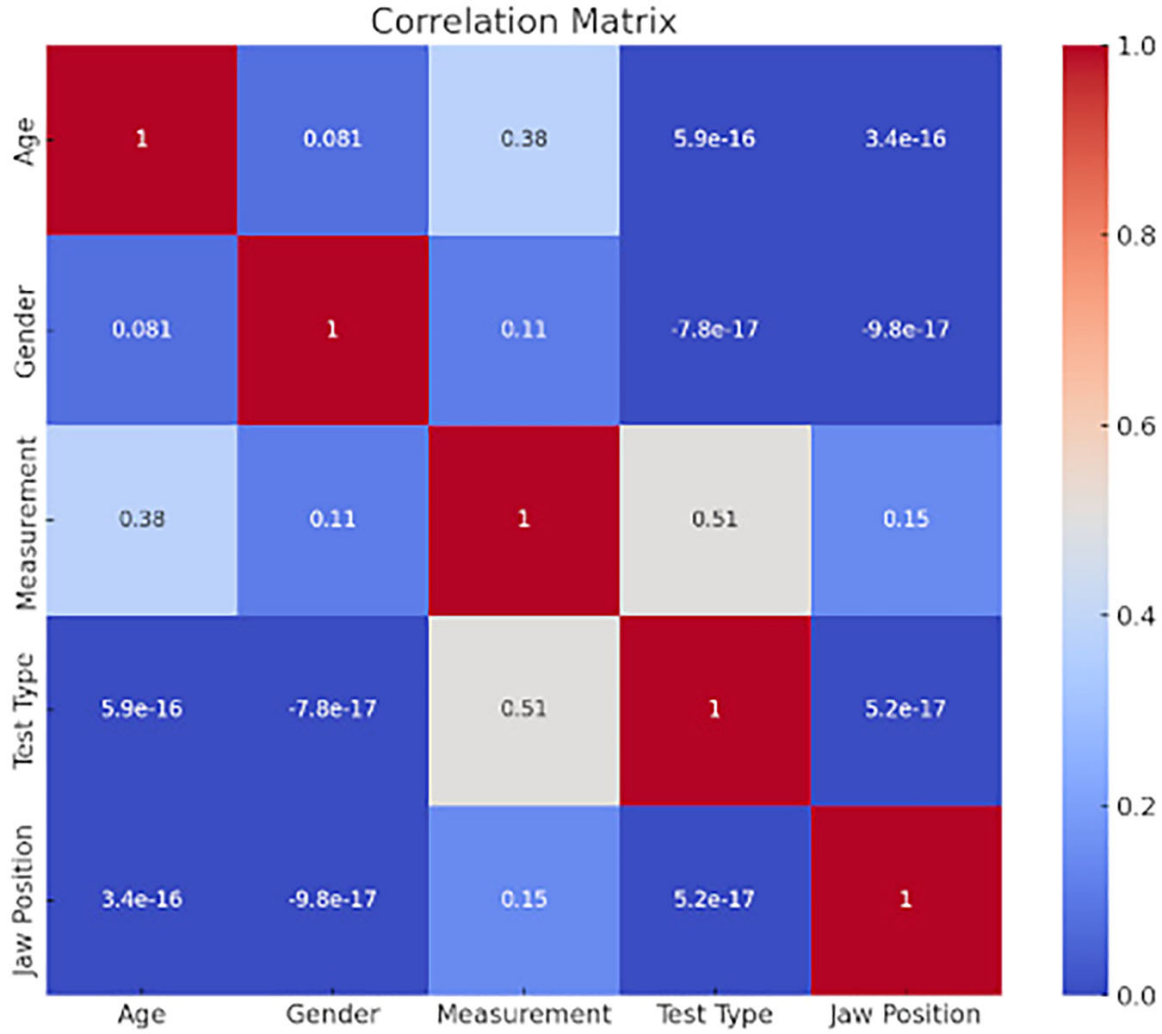
Correlation matrix of variables related to occlusal splint treatment outcomes.

This correlation matrix illustrates the relationships between age, gender, jaw position, measurement outcomes (accommodative capacity and ocular convergence), and test type (occlusal splint treatment) in individuals with TMDs. The color gradient from blue to red represents the strength and direction of the correlations, with blue indicating negative correlations, red indicating positive correlations, and white indicating weak or no correlation. The results include a moderate positive correlation (0.51) between test type and measurement outcomes, suggesting a measurable effect of occlusal splint treatment on accommodative capacity and ocular convergence. On the other hand, the correlation between test type and jaw position is practically zero (5.2e-17), indicating no significant effect on jaw position. The matrix provides insights into the dynamics between the TMJ and the visual apparatus, supporting the hypothesis that occlusal splint treatment can influence visual outcomes in individuals with TMDs (Fig. 2).

Examined was the relationship between time points (T0, T1, T2), measurements, and other variables such as age, gender, and jaw position. Recognized for its efficacy in regression and classification tasks, Random Forest, an ensemble learning method, was utilized. This method proved advantageous in capturing non-linearities and interactions between variables without necessitating an explicit model specification.

#### Applied to the data was the Random Forest model, yielding the following performance metrics

MSE was 3.21, which shows how far the predicted values were from the actual values. This indicates a moderate level of prediction error—not perfect, but not too bad either.

*R*² value was 0.55, meaning the model could explain 55% of the variations in the data. This suggests the model had a moderate ability to predict the results based on the given information.

**Fig. 3:**
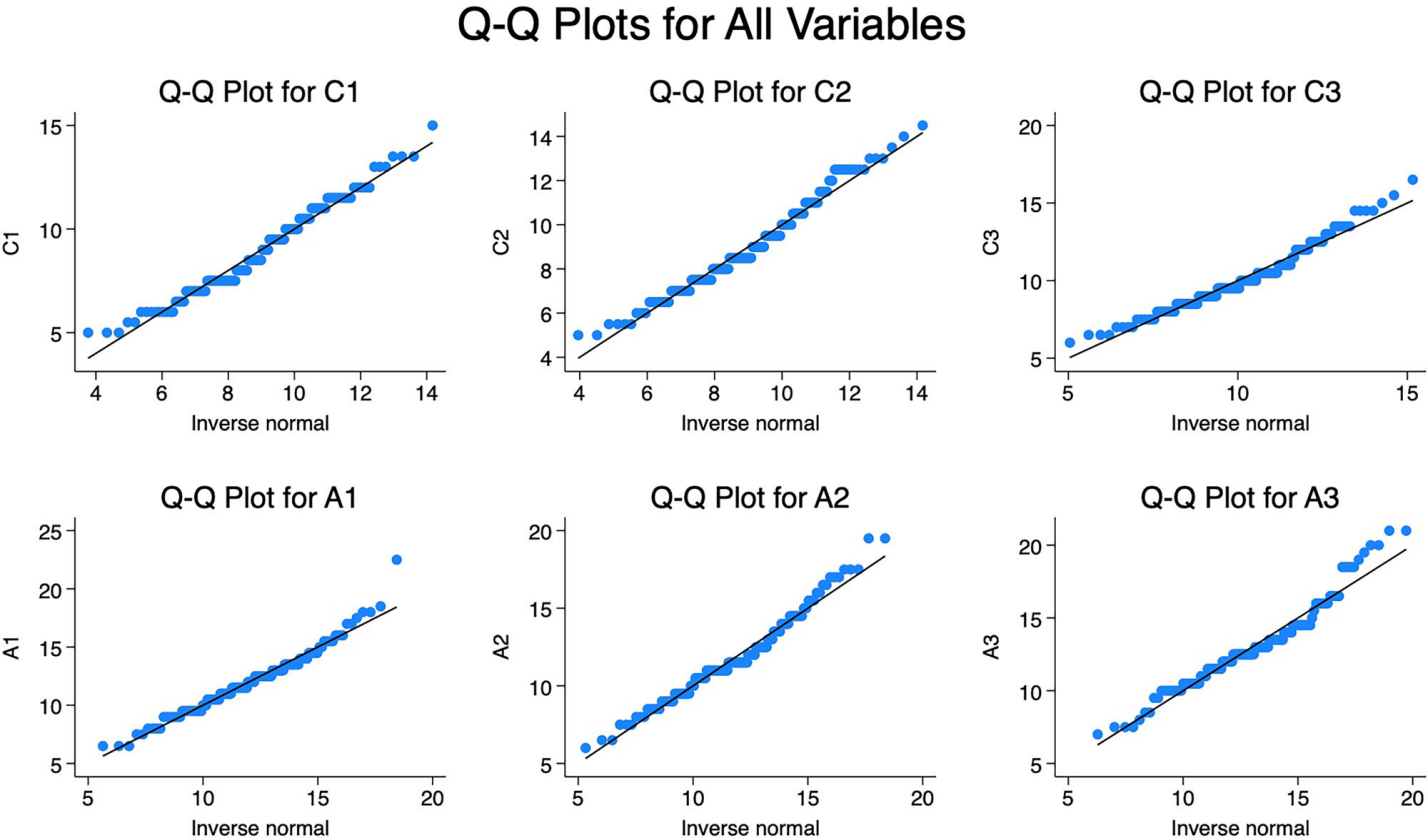
Normal distribution.

These findings suggest that the data was non-normally distributed (Fig. 3). The results of the Random Forest model suggest that it effectively captured a significant portion of the complexity and non-linear relationships present in the data. There needs a cautious interpretation of the results, particularly in the context of the specific research questions and data characteristics.

## Discussion

Based on the neuroanatomical and clinical observations reported in the existing literature, we thought it could be interesting to assess whether the visual apparatus could also be influenced by the treatment of stomatognathic disorders. So, our research tried to understand if an occlusal splint, normally used for the treatment of some TMDs, could also influence visual capacities (in particular, we focalized our attention on accommodation and ocular convergence).

While the stomatognathic system’s influence on dental and ophthalmic sciences is acknowledged, limited research has explored the therapeutic impact on vision. The novelty lies in bridging disciplinary boundaries and proposing innovative therapeutic strategies to enhance our understanding of the symbiotic relationship between the TMJ and the visual apparatus in the context of TMD.

Firstly, the comparison of the average measurements from both the accommodation and the convergence test showed how an increasing distance between the dental arches (open mouth position) generates progressively worse results. While maximum occlusal contact (obtained at the position of maximum intercuspation) can led to the best measurement results. In particular for the accommodation, a higher value indicates a lower accommodative capacity; while a higher convergence value indicates a lower neuromuscular capacity [29–34].

Moreover, accommodation and ocular convergence, after 6 months of follow-up, we obtained better results at maximum intercuspation and rest position, but only the convergence values at maximum intercuspation resulted statistical significance (*p* < 0.01). A possible explanation of these results could be, that, nociceptive stimuli due to inflammation of the temporomandibular system could trigger nervous dysfunctions involving the extrinsic ocular musculature: given that the proprioception of the extrinsic ocular muscles appears to be connected with the trigeminal sensorial and proprioceptive structures, the extrinsic muscular imbalances of the eye could influence the trigeminal afferent and in the same way the nociceptive stimuli and the trigeminal proprioception could modulate the extrinsic tone’s muscle of the eye [9, 10].

Clinically, these concepts are important because it’s possible to hypothesize that the OST, leading to an improvement in TMDs and relative pain, could minimize any alterations in nerve conduction, which, as we have said, could affect visual abilities. In fact the main occlusal splint functions are to change the working length of the masticatory muscles during clenching due to the variation in the vertical dimension, reducing tension, muscle fatigue, and pain. Furthermore, it should favor a decompression of the joint surfaces burdened by overload, optimizing TMJ’s function [9, 10].

A recent systematic review and meta-analysis compared OST and manual therapy (MT) for treating TMJ disorders (TMDs). Analyzing nine studies with 426 patients, the review found that both therapies effectively reduce pain and improve quality of life. However, MT showed superior efficacy in improving TMJ function, increasing maximal mouth opening, and reducing disability. While MT may not be significantly better than OST for pain relief and health-related quality of life, OST was suggested to be more effective for myogenic TMDs, though with weak supporting evidence. The study recommends a combined approach of OST and MT for TMD treatment but calls for further research to strengthen these findings [27].

In our study, multiple statistical tests were performed to assess changes in accommodative capacity and ocular convergence across different time points and jaw positions. It is important to acknowledge that performing multiple comparisons increases the likelihood of obtaining at least one statistically significant result by chance (Type I error). The significant result observed in the convergence test at maximum intercuspation (Jaw Position 1, *p* = 0.034) should therefore be interpreted with caution. While this result suggests a potential influence of OST on convergence, it does not necessarily confirm a causal relationship due to the possibility of false positives arising from multiple testing. Also, in addition multiple statistical tests help to understand the results comprehensively and direct future study designs, sample sizes to be included to have a more cohesive, unbiased approach, validating the results.

## Conclusion

In conclusion, our study results found that there are some effects on visual acuity during occlusal splint treatment. More specifically, there was a statistically significant result for the convergence outcome at maximum intercuspation. The findings should be interpreted with great caution, and following factors, such as a small sample size, valid visual tests, and operator experience, should be considered. There is a need for well-designed longitudinal studies and clinical trials.

From this perspective, we can explore whether the TMD and visual systems might also impact the postural system, like head position. This would help us see if treating one system (TMD, visual, or postural) could benefit not just those system, but others as well.

### Clinical relevance

Although the statistical significance observed in the convergence test at maximum intercuspation suggests a measurable change, the effect size and clinical significance must be carefully considered. Given that other jaw positions did not show statistically significant improvements, it is uncertain whether this effect translates into meaningful functional improvement for patients. Future studies with larger sample sizes and clinically validated thresholds will be necessary to determine whether the observed changes have practical implications for visual function in TMD patients.

### Future research

Clinically, the findings suggest that OST may influence visual function, but more good, designed studies are needed to validate these results with more sample sizes. Future research should employ larger, well-controlled trials, cohort studies to assess the extent of this relationship. Additionally, incorporating advanced statistical methods, such as hierarchical modeling or false discovery rate control, may provide deeper insights into the interplay between occlusal treatment and vision.

## Data Availability

The data supporting the findings of this study are available upon reasonable request from the corresponding author.
